# A Retrospective Administrative Database Analysis of Suicide Attempts and Completed Suicide in Patients With Chronic Pancreatitis

**DOI:** 10.3389/fpsyt.2018.00147

**Published:** 2018-04-18

**Authors:** Chien-Hua Chen, Cheng-Li Lin, Chung-Y. Hsu, Chia-Hung Kao

**Affiliations:** ^1^Digestive Disease Center, Show-Chwan Memorial Hospital, Changhua, Taiwan; ^2^Digestive Disease Center, Changbing Show-Chwan Memorial Hospital, Lukang, Taiwan; ^3^Department of Food Science and Technology, Hungkuang University, Taichung, Taiwan; ^4^Chung Chou University of Science and Technology, Yuanlin, Taiwan; ^5^Management Office for Health Data, China Medical University Hospital, Taichung, Taiwan; ^6^College of Medicine, China Medical University, Taichung, Taiwan; ^7^Graduate Institute of Clinical Medical Science, China Medical University, Taichung, Taiwan; ^8^Graduate Institute of Clinical Medical Science, School of Medicine, College of Medicine, China Medical University, Taichung, Taiwan; ^9^Department of Nuclear Medicine and PET Center, China Medical University Hospital, Taichung, Taiwan; ^10^Department of Bioinformatics and Medical Engineering, Asia University, Taichung, Taiwan

**Keywords:** chronic pancreatitis, suicide, comorbidity, cohort study, population based

## Abstract

**Background:** The actual incidence rate of suicide attempt and the suicide-related fatality rate (completed suicide) in patients with chronic pancreatitis (CP) have not been mentioned in the literature.

**Methods:** We conducted a nationwide population-based cohort study by analyzing data from Taiwan's National Health Insurance Research Database (NHIRD) to compare the rate of suicide attempt between a CP cohort and a non-CP cohort. For the study cohort, we identified 17,733 patients (age ≥ 20 years) diagnosed as having CP between 2000 and 2010 from the NHIRD in Taiwan. Beneficiaries with no history of CP were matched with the study cohort at a 2:1 ratio according to age, sex, and index date. To determine the incidence of suicide, all patients were followed until the end of 2011 or until their withdrawal from the Taiwan National Health Insurance program.

**Results:** Patients with CP had an increased risk of suicide attempt, compared with those without CP (adjusted hazard ratio [aHR] = 2.72, 95% confidence interval [CI] = 1.69–4.37). The suicide-related fatality in the CP cohort was higher than that in the non-CP cohort, but the difference was not statistically significant (aHR = 1.21, 95% CI = 0.39–3.78).

**Conclusion:** Our population-based cohort study reveals a close association between CP and subsequent suicide attempt. Compared with the non-CP cohort, the suicide-related fatality was higher in the CP cohort, although the result was not statistically significant. These findings necessitate surveying patients with CP and providing psychological support to prevent suicide.

## Introduction

Suicide is generally defined as an intentional act to cause one's own injury or death although there is no single accepted definition [1]. Nevertheless, suicide can lead to no injuries, injuries, or death. Suicide attempt means an action through which an individual intentionally hurt oneself, whatever the degree of lethality and the recognition of the underlying motivation [2]. Moreover, completed suicide means the death attributable to the suicide behaviors [3]. Therefore, completed suicide will be one of the results of suicide attempt in our study. More than 800,000 people die by suicide annually worldwide, representing ~1.4% of the global population [4]. The reported average rate of suicide attempt in Taiwan is 16.5 per 100,000 population between 2003 and 2015 [5]. However, the reported rate of completed suicide has increased from 7.6 per 100,000 population in 1995, peaked to 19.3 per 100,000 population in 2006, and then declined to 15.7 per 100,000 population in 2015 [6]. Attempted suicide and completed suicide rates vary substantially with respect to sex, age, race, urbanization level, socioeconomic status, and other comorbidities of psychiatric or physiological disorders [7]. Suicide can occur at any age, and reports have revealed that there are 20–30 times more suicide attempts than completed suicides in some areas [8, 9]. Therefore, determining individuals who are at a high risk of attempting suicide is a public health priority, to provide adequate treatment and management programs for such people [10, 11].

Chronic pancreatitis (CP) causes irreversible damage to the pancreas from chronic progressive inflammation, and it mainly results in physiological disorders such as endocrine or exocrine insufficiency [12]. The causes of CP are deemed to be multifactorial and interrelated; however, heavy alcohol consumption is deemed to be the most common cause, and the literature states that alcohol is involved in ~70–80% of CP cases [13]. Endocrine or exocrine functions are complex, and obtaining pathological findings is inconvenient in clinical practice; therefore, definitive diagnosis of CP mainly depends on imaging findings of pancreatic calcifications, pancreatic stones, and pancreatic duct stenosis or dilatation using ultrasonography, computed tomography, or magnetic resonance imaging (MRI) [14]. However, because of the increasing rates of alcohol consumption, more extensive utilization of diagnostic imaging techniques, and greater awareness of CP, the incidence of CP has been reported to have slightly increased globally [15]. The reported rate of alcoholism in Taiwan is 2.40% for young adults (aged 18–39 years) and 2.27% for middle-aged adults (aged 40–64 years) based on 2009 Taiwan National Health Insurance Interview Survey [16]. The reported crude and adjusted incidence rates of acute pancreatitis in Taiwan is 56.9 and 42.8 per 100,000 population in 2005, respectively; nevertheless, the incidence of chronic pancreatitis in Taiwan has never been mentioned in the literature. However, the reported prevalence of CP in China has increased from 3.08 per 100,000 population in 1996 to 13.52 per 100,000 population in 2003 [17, 18].

The symptoms of CP vary and are unpredictable, but abdominal pain remains the most common symptom and may present as an acute exacerbation or as a constant intractable pain [19]. The methods of managing pain for CP followed in the order of priority includes medication with pancreatic enzyme supplementation or analgesics, endoscopic lithotripsy or decompression of the pancreatic duct, and surgical resection of the pancreatic head or decompression of the pancreatic duct [20]. However, there are only 95 patients of the non-cancer pain patients receiving long-term usage of narcotic analgesic for CP in Taiwan from 2003 to 2012 [21]. The rate of opioid dependence in Taiwan could not be accurately estimated, but only 983 individuals participated opioid agonist treatment in 2006 [22]. Although CP occurs in only a small percentage of the population, with a reported prevalence of 3–42/100,000 population, it has been considered to consume a disproportionate amount of resources [23, 24]. CP causes a profound decrease in physical quality of life (QOL) and has a clinically significant negative effect on mental QOL [25]. However, the poor QOL associated with CP has no relation to the socioeconomic status of a patient, their drinking or smoking habits, or other common medical comorbidities [25]. Nevertheless, most clinicians typically focus on the physiological influences of CP and overlook its psychological impacts since the Asia-Pacific consensus report on the management of CP only focused on pain relief, diabetes, and steatorrhea without mentioning the psychological support [20].

Suicide is one of the leading causes of death or disability; therefore, in addition to providing physiological care, health care providers must determine the risk of suicide in patients with CP through evidence-based surveys. Chen et al. has reported that disease of the pancreas is an independent risk factor for completed suicide in psychiatric patients [26]. Nevertheless, the aforementioned study did not specify chronic pancreatitis, only targeted the population with previous psychiatric diagnoses, and this study did not discuss the risk of suicide attempt. To our knowledge, no population-based cohort studies have reported suicide attempts and the suicide-related fatality in patients with chronic pancreatitis. We hypothesized that CP is associated with an increased risk of suicide attempt and the suicide-related fatality. We conducted a nationwide population-based cohort study by analyzing data from Taiwan's National Health Insurance Research Database (NHIRD) to assess the aforementioned association and to compare the rates of suicide attempt and suicide-related fatality between a CP cohort and a non-CP cohort.

## Methods

### Data source

We used the NHIRD of the National Health Insurance (NHI) program in Taiwan to conduct this retrospective nationwide cohort study [27]. The NHI program was established by the Taiwanese government on March 1, 1995, and it covers more than 99.5% of the 23.74 million residents in Taiwan. Health care data of all patients enrolled in the NHI program are stored in databases. The National Health Research Institutes (http://nhird.nhri.org.tw/), a government-established non-profit organization for medical research, maintains the NHIRD (http://nhird.nhri.org.tw/en/index.html) [28, 29]. All data within the NHIRD are officially encrypted and researchers can utilize them for medical research after formal application and approval. All disease codes were established based on the 2001 International Classification of Diseases, Ninth Revision, Clinical Modification (ICD-9-CM).

### Ethics statement

The NHIRD encrypts patient personal information to protect privacy and provides researchers with anonymous identification numbers associated with relevant claims information, including sex, date of birth, medical services received, and prescriptions. Therefore, patient consent is not required to access the NHIRD. This study was approved to fulfill the condition for exemption by the Institutional Review Board (IRB) of China Medical University (CMUH-104-REC2-115-CR2). The IRB also specifically waived the consent requirement.

### Patients

The study cohort contained patients aged ≥20 years with newly diagnosed CP (ICD-9-CM: 577.1) between January 1, 2000, and December 31, 2010, who were identified from the inpatient claims with the diagnostic codes linkage for hospitalization or visiting emergency department; the index date was defined as the date of CP diagnosis. The control cohort comprised individuals without a history of CP who were randomly selected from the NHIRD and matched with the study cohort at a 2:1 ratio. Patients in the non-CP (ICD-9-CM: 577.1) cohort were frequency matched according to age (in 5-year intervals), sex, and index year. Both the CP and non-CP cohorts excluded patients with a history of pancreatic cancer (ICD-9-CM 157) or a history for suicide attempt (ICD-9-CM codes E950–E959), or those with incomplete information relating to age or sex at enrollment. The codes for suicide behaviors (ICD-9-CM codes E950–E959) relate to liquid or solid poisoning (ICD-9-CM code E950), charcoal burning and poisoning by gases (ICD-9-CM code E952), hanging (ICD-9-CM code E953), cutting/piercing (ICD-9-CM code E956), jumping from high places (ICD-9-CM code E957), and others (ICD-9-CM codes E951, E954, E955, E958, and E959). Each patient was surveyed from the index date to the incidence of suicide attempt, death, withdrawal from the NHI program, or the end of 2011. Emigration from Taiwan and death are the main reasons for beneficiaries withdrawing from the NHI program. Cause-specific and non-cause-specific deaths were included in the analysis if they could be identified, but patients were censored if the causes of death could not be identified. In addition, the index date was set based on the month and day of the same index year.

Comorbidities in our study were as follows: schizophrenic disorders (ICD-9-CM code 295), depression (ICD- 9-CM codes 296.2, 296.3, 300.4, and 311), alcohol-related illness (ICD-9-CM codes 291, 303, 305, 571.0, 571.1, 57P1.2, 571.3, 790.3, A215, and V11.3), anxiety (ICD-9-CM code 300.00), mental disorders (ICD-9-CM codes 290–319), insomnia (ICD-9-CM code 780.52), acute pancreatitis (ICD-9-CM code 577.0), drug abuse(ICD-9-CM codes 304 and 305), psychalgia (ICD-9-CM code 307.8), chronic obstructive pulmonary disease (ICD-9-CM codes 491, 492, 496), chronic kidney disease (ICD-9-CM codes 585), diabetes mellitus (ICD-9-CM code 250), cardiovascular disease(ICD-9-CM codes 410–414, 428, 430–438, 440–448), hyperlipidemia (ICD-9-CM code 272), and hypercalcemia (ICD-9-CM code 275.42). The city districts and townships of Taiwan were classified into seven urbanization levels based on population density (people/km^2^), proportion of residents with higher education, elderly, and agricultural population, and the number of physicians per 100,000 people in each area [30]. The areas with a higher population density and socioeconomic status were labeled as level 1, whereas we grouped these areas between levels 4 and 7 into the level four group since few people lived in the rural areas. The employees with indoor works such as public institutional workers, educators, or administrative personnel in business and industries were classified as office workers. The employees with longer hours of outdoor works such as fishermen, farmers, or industrial laborers were classified as laborers. The subjects who were primarily retired, unemployed, and low-income populations classified as those with other occupations [31]. The beneficiaries should pay the fee according to their income levels, and all the beneficiaries should register their places of residence and occupation categories for NHI program. All the pre-existing comorbidities, place of residence, and socioeconomic status would be redefined as long as they were updated in the NHIRD prior to the endpoint.

### Statistical analysis

The chi-squared test was used to compare the distributions of age, sex, monthly income, urbanization level, occupation category, and comorbidities between the CP and non-CP cohorts. The Student t test was used to compare the mean ages (standard deviations, SDs), frequency of medical visits, and follow-up periods (SDs) between the two cohorts. The Kaplan–Meier method was used to compare the cumulative incidence of suicide attempt and survival between the two cohorts, and the log-rank test was used to examine the differences. The incidence-density rates of suicide attempt were estimated by dividing the number of suicide events by the number of person-years for each risk factor and subsequently stratifying these by age, sex, monthly income, urbanization level, occupation category, and comorbidities. The risk of CP-associated suicide attempt and suicide-related fatality was assessed using univariable and multivariable Cox proportional hazards regression models. The corresponding hazard ratios (HRs) and 95% confidence intervals (CIs) were estimated using a Cox model adjusted for age, monthly income, urbanization level, occupation category, frequency of medical visits, and comorbidities of depression, alcohol-related illness, anxiety, mental disorders, insomnia, acute pancreatitis, psychalgia, drug abuse, chronic obstructive pulmonary disease, diabetes mellitus, cardiovascular disease, and hyperlipidemia. The death event was deemed as a competing event to estimate the sub-HRs (SHRs) and 95% CIs by using extensions of the standard univariable and multivariable Cox proportional hazard regression models. We used SAS Version 9.4 (SAS Institute, Cary, NC, USA) for data analyses, and a two-tailed *P*-value of <0.05 was considered statistically significant.

## Results

This study examined CP and non-CP cohorts comprising 17,733 and 35,466 patients, respectively (Table 1). The two cohorts were well-matched for age, sex, and index date. The mean ages of patients in the CP and non-CP cohorts were 48.6 ± 15.2 and 48.3 ± 15.5 years, respectively. Most patients were younger than 49 years (62.8%) and men (82.6%). The patients in the CP cohort registered a higher frequency of medical visits than did those in the non-CP cohort. Comorbidities in the cohorts were sorted according to frequency of occurrence as follows: history of acute pancreatitis (68.9%), alcohol-related illness (52.6%), diabetes mellitus (46.8%), mental disorders (33.3%), hyperlipidemia (32.0%), cardiovascular disease (27.3%), insomnia (13.7%), depression (10.7%), chronic obstructive pulmonary disease (10.5%), chronic kidney disease (6.16%), drug abuse (5.29%), anxiety (3.78%), schizophrenic disorders (0.95%), psychalgia (0.83%), and hypercalcemia (0.04%). Our study showed no linear correlation between CP and the levels of monthly income, urbanization, or occupation category.

**Table 1:** Comparisons of demographic characteristics and comorbidities in patients with and without chronic pancreatitis.

	**Chronic pancreatiti**		***p*-value**
	**No**	**Yes**	
	**(*N* = 35,466)**	**(*N* = 17,733)**	
Gender			0.99
Women	6, 172(17.4)	3, 086(17.4)	
Men	29, 294(82.6)	14, 647(82.6)	
Age stratified			0.99
≤49	22, 276(62.8)	11, 138(62.8)	
50–64	7, 124(20.1)	3, 562(20.1)	
65+	6, 066(17.1)	3, 033(17.1)	
Age, mean ± SD^a^	48.3(15.5)	48.6(15.2)	0.01
Monthly income^†^			<0.001
<15,000	7, 674(21.6)	4, 869(27.5)	
15,000–19,999	15, 581(43.9)	9, 288(52.4)	
≥20,000	12, 211(34.4)	3, 576(20.2)	
Urbanization level^‡^			<0.001
1 (highest)	10, 126(28.6)	3, 648(20.6)	
2	10, 900(30.7)	5, 356(30.2)	
3	6, 253(17.6)	2, 965(16.7)	
4 (lowest)	8, 187(23.1)	5, 764(32.5)	
Occupation category^&^			<0.001
Office worker	20, 068(56.6)	7, 446(42.0)	
Laborer	11, 629(32.8)	7, 466(42.1)	
Other	3, 769(10.6)	2, 821(15.9)	
Frequency of medical visits/per year, mean ± SD^a^	0.57(2.02)	7.01(6.83)	<0.001
Comorbidity			
Schizophrenic disorders	261(0.74)	168(0.95)	0.01
Depression	339(0.96)	1, 898(10.7)	<0.001
Alcohol-related illness	447(1.26)	9, 333(52.6)	<0.001
Anxiety	168(0.47)	670(3.78)	<0.001
Mental disorders	894(2.52)	5, 908(33.3)	<0.001
Insomnia	504(1.42)	2, 433(13.7)	<0.001
Acute pancreatitis	377(1.06)	12, 212(68.9)	<0.001
Psychalgia	49(0.14)	148(0.83)	<0.001
Drug abuse	66(0.19)	938(5.29)	<0.001
Chronic obstructive pulmonary disease	1, 279(3.61)	1, 866(10.5)	<0.001
Chronic kidney disease	470(1.33)	1, 092(6.16)	<0.001
Diabetes mellitus	2, 879(8.12)	8, 296(46.8)	<0.001
Cardiovascular disease	4, 169(11.8)	4, 845(27.3)	<0.001
Hyperlipidemia	1, 492(4.21)	5, 680(32.0)	<0.001
Hypercalcemia	1(0.00)	7(0.04)	0.001

*Chi-squared Test*,

a *t-test*.

† *New Taiwan Dollar (NTD), 1 NTD is equal to 0.03 USD*.

‡ *The urbanization level was determined by dividing the population density of the residential area into four levels, with level 1 being the most urbanized and level 4 the least urbanized*.

& *Other occupation categories included those who were primarily retired, unemployed, and low-income populations*.

**Figure 1:**
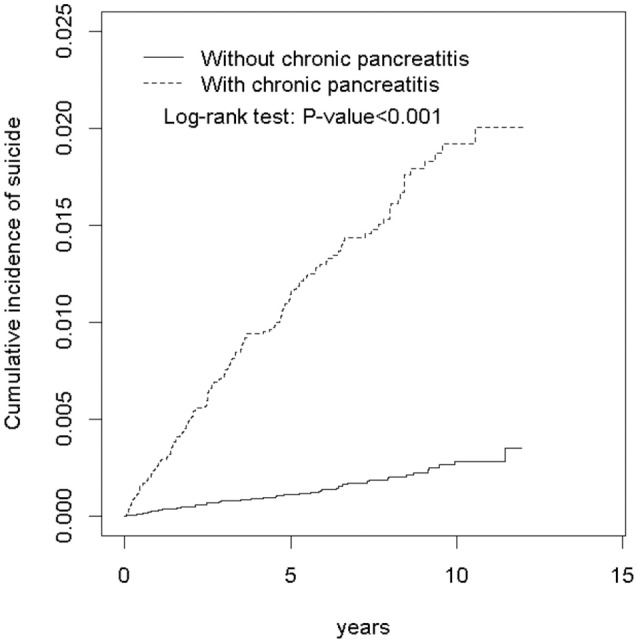
Cumulative incidence of suicide attempt for individuals with and without chronic pancreatitis.

Figure 1 presents a higher cumulative incidence of suicide attempt in the CP cohort than in the non-CP cohort (log-rank test, *P* < 0.001). The CP and non-CP cohorts had average follow-up durations of 4.07 ± 3.35 and 5.58 ± 3.22 years, respectively.

Table 2 presents the incidence of and risk factors for suicide attempt. The risk of suicide attempt was not related to monthly income or to the occupation category. The overall incidence density rates of suicide attempt for the CP and non-CP cohorts were 21.2 and 2.53 per 10,000 person-years, respectively. Compared with the non-CP cohort, those with CP were associated with an increased risk of suicide attempt (adjusted HR [aHR] = 2.72, 95% CI = 1.69–4.37), after adjustment for age, monthly income, urbanization level, occupation category, frequency of medical visits/per year, and comorbidities of depression, alcohol-related illness, anxiety, mental disorders, insomnia, acute pancreatitis, psychalgia, drug abuse, chronic obstructive pulmonary disease, diabetes mellitus, cardiovascular disease and hyperlipidemia. Compared with patients living in areas of level 1 urbanization, the risk of suicide attempt was 1.69-fold higher for those living in areas of level 3 urbanization (95% CI = 1.08–2.65) and 2.01-fold higher for those living in areas of level 4 urbanization (95% CI = 1.34–3.01). Among the comorbidities, multivariable analysis identified only depression (aHR = 10.1, 95% CI = 6.62–15.4) and mental disorders (aHR = 2.09, 95% CI = 1.32–3.31) as independent risk factors for suicide attempt.

**Table 2:** Incidence and risk factors for suicide attempt.

**Variable**	**Event**	**PY**	**Rate^#^**	**Crude HR (95% CI)**	**Adjusted HR^$^ (95% CI)**
**CHRONIC PANCREATITIS**					
No	54	213,812	2.53	1.00	1.00
Yes	183	86,325	21.2	8.26 (6.10, 11.2)^***^	2.72 (1.69, 4.37)^***^
**AGE GROUP, YEARS**					
≤49	185	197,862	9.35	1.99 (1.34, 2.96)^***^	1.29 (0.85, 1.96)
50–64	28	59,008	4.75	1.00	1.00
65+	24	43,268	5.55	1.15 (0.67, 1.98)	1.46 (0.82, 2.58)
**GENDER**					
Women	35	51,601	6.78	1.20 (0.84, 1.71)	–
Men	202	248,536	8.13	1.00	1.00
**MONTHLY INCOME^†^**					
<15,000	68	65,852	10.3	2.31 (1.57, 3.39)^***^	1.17 (0.73, 1.89)
15,000–19,999	127	138,658	9.16	2.07 (1.46, 2.93)^***^	1.19 (0.80, 1.76)
≥20,000	42	95,627	4.39	1.00	1.00
**URBANIZATION LEVEL^‡^**					
1 (highest)	33	79,442	4.15	1.00	1.00
2	63	92,067	6.84	1.64 (1.08, 2.50)^*^	1.35 (0.88, 2.07)
3	46	51,597	8.92	2.14 (1.37, 3.34)^***^	1.69 (1.08, 2.65)^*^
4 (lowest)	95	77,031	12.3	2.95 (1.99, 4.39)^***^	2.01 (1.34, 3.01)^***^
**OCCUPATION CATEGORY^&^**					
Office worker	95	157,374	6.04	1.00	1.00
Laborer	99	107,883	9.18	1.52 (1.15, 2.01)^**^	1.07 (0.77, 1.49)
Other	43	34,881	12.3	2.02 (1.41, 2.90)^***^	1.24 (0.77, 1.98)
**COMORBIDITY SCHIZOPHRENIC**					
No	234	298,458	7.84	1.00	1.00
Yes	3	1,680	17.9	2.25 (0.72, 7.02)	–
**DEPRESSION**					
No	211	295,723	7.14	1.00	1.00
Yes	26	4,414	58.9	7.95 (5.28, 12.0)^***^	10.1 (6.62, 15.4)^***^
**ALCOHOL-RELATED ILLNESS**					
No	117	260,052	4.50	1.00	1.00
Yes	120	40,086	29.9	6.53 (5.06, 8.43)^***^	1.22 (0.79, 15.4)
**ANXIETY**					
No	228	298,018	7.65	1.00	1.00
Yes	9	2,120	42.5	5.43 (2.79, 10.6)^***^	1.07 (0.65, 1.76)
**MENTAL DISORDERS**					
No	204	279,737	7.29	1.00	1.00
Yes	33	20,400	16.2	2.15 (1.49, 3.10)^***^	2.09 (1.32, 3.30)^***^
**INSOMNIA**					
No	214	294,396	7.27	1.00	1.00
Yes	23	5,742	40.1	5.27 (3.42, 8.11)^***^	0.85 (0.60, 1.22)
**ACUTE PANCREATITIS**					
No	121	251,032	4.82	1.00	1.00
Yes	116	49,106	23.6	4.82 (3.73, 6.22)^***^	0.96 (0.65, 1.42)
**PSYCHALGIA**					
No	234	298,946	7.83	1.00	1.00
Yes	3	1,192	25.2	3.26 (1.04, 10.2)^*^	0.55 (0.17, 1.81)
**DRUG ABUSE**					
No	210	294,777	7.12	1.00	1.00
Yes	27	5,360	50.4	6.99 (4.68, 10.4)^***^	0.80 (0.52, 1.24)
**CHRONIC OBSTRUCTIVE PULMONARY DISEASE**					
No	217	284,650	7.62	1.00	1.00
Yes	20	15,487	12.9	1.67 (1.05, 2.64)^*^	0.66 (0.41, 1.09)
**CHRONIC KIDNEY DISEASE**					
No	228	293,134	7.78	1.00	1.00
Yes	9	7,004	12.9	1.61 (0.83, 3.13)	–
**DIABETES MELLITUS**					
No	152	241,470	6.29	1.00	1.00
Yes	85	58,668	14.5	2.28 (1.75, 2.97)^***^	0.78 (0.58, 1.04)
**CARDIOVASCULAR DISEASE**					
No	184	252,916	7.28	1.00	1.00
Yes	53	47,222	11.2	1.53 (1.13, 2.08)^**^	0.74 (0.52, 1.04)
**HYPERLIPIDEMIA**					
No	162	260,213	6.23	1.00	1.00
Yes	75	39,924	18.8	3.01 (2.29, 3.96)^***^	0.90 (0.66, 1.22)
**HYPERCALCEMIA**					
No	237	300,101	7.90	1.00	1.00
Yes	0	37	0.00	–	–

*CI, confidence interval; HR, hazard ratio; PY, person-years*.

# *Incidence rate per 10,000 person-years*.

$ *Multivariable analysis included age, monthly income, urbanization level, occupation category, frequency of medical visits/per year, and comorbidities of depression, alcohol-related illness, anxiety, mental disorders, insomnia, acute pancreatitis, psychalgia, drug abuse, chronic obstructive pulmonary disease, diabetes mellitus, cardiovascular disease, and hyperlipidemia*.

† *New Taiwan Dollar (NTD), 1 NTD is equal to 0.03 USD*.

‡ *Urbanization level was determined by dividing the population density of residential areas into four levels, with level 1 being the most urbanized and level 4 the least urbanized*.

& *Other occupation categories included those who were primarily retired, unemployed, and low-income populations*.

* *P < 0.05*,

** *P < 0.01*,

*** *P < 0.001*.

Table 3 presents a comparison of the incidence densities of suicide attempt in the CP and non-CP cohorts according to demographic characteristics and comorbidities. The contribution of CP to the age-specific relative risk of suicide was greater in younger patients (≤49 years: aHR = 3.99, 95% CI = 2.16–7.35; and 50–64 years: aHR = 3.79, 95% CI = 1.22–11.8) than in non-CP cohort. Furthermore, the contribution of CP to the sex-specific relative risk of suicide was greater in men (men, aHR = 2.77, 95% CI = 1.59–4.82) than in in non-CP cohort. Monthly income-specific data show that compared with non-CP cohort, patients with CP exhibited higher risks for suicide attempt in the monthly income category of 15,000–19,999 NTD (aHR = 2.67, 95% CI = 1.39–5.14). The urbanization level-specific analysis revealed that the CP to non-CP aHRs for suicide attempt were significantly higher in the categories of second highest urban areas. The occupation-specific CP to non-CP suicide attempt risks were higher in office worker. The contribution of CP to the relative risk of suicide attempt was greater in patients without or with comorbidities (aHR = 4.31, 95% CI = 2.17–8.53; aHR = 2.26, 95% CI = 1.34–3.79) than in those without CP. Compared with the non-CP cohort, the CP cohort had a higher risk of suicide attempt (aHR = 7.19, 95% CI = 2.97–17.4) in the first year of follow-up. Moreover, the risk of suicide attempt in the CP cohort was still significantly higher than that in the non-CP cohort after 3 years of follow-up (aHR = 2.32, 95% CI = 1.26–4.26).

**Table 3:** Incidence and hazard ratios of suicide attempt for individuals with and without chronic pancreatitis stratified by demographics and comorbidities.

	**Chronic pancreatitis**							
	**No**			**Yes**				
**Outcome**	**Event**	**PY**	**Rate^#^**	**Event**	**PY**	**Rate^#^**	**Crude HR (95% CI)**	**Adjusted HR^$^ (95% CI)**
**AGE GROUP, YEARS**								
≤49	31	139,529	2.22	154	58,333	26.4	11.6(7.91, 17.1)^***^	3.99(2.16, 7.35)^***^
50–64	7	42,578	1.64	21	16,430	12.8	7.71(3.27, 18.2)^***^	3.79(1.22, 11.8)^*^
65+	16	31,706	5.05	8	11,562	6.92	1.36(0.58, 3.19)	0.65(0.20, 2.11)
**GENDER**								
Women	10	36,745	2.72	25	14,856	16.8	6.19(2.97, 12.9)^***^	1.92(0.70, 5.23)
Men	44	177,067	2.48	158	71,469	22.1	8.72(6.24, 12.2)^***^	2.77(1.59, 4.82)^***^
**MONTHLY INCOME^†^**								
<15,000	14	43,463	3.22	54	22,389	24.1	7.13(3.96, 12.8)^***^	2.12(0.90, 5.01)
15,000–19,999	29	93,102	3.11	98	45,556	21.5	6.89(4.55, 10.4)^***^	2.67(1.39, 5.14)^**^
≥20,000	11	77,247	1.42	31	18,380	16.9	11.9(5.99, 23.8)^***^	3.26(1.00, 10.7)
**URBANIZATION LEVEL^‡^**								
1 (highest)	9	61,823	1.46	24	17,618	13.6	9.35(4.34, 20.1)^***^	0.85(0.19, 3.84)
2	11	65,691	1.67	52	26,377	19.7	11.5(5.98, 22.0)^***^	5.33(2.18, 13.0)^***^
3	15	36,676	4.09	31	14,921	20.8	5.03(2.72, 9.33)^***^	1.68(0.57, 4.94)
4 (lowest)	19	49,622	3.83	76	27,409	27.7	7.15(4.32, 11.8)^***^	2.21(0.99, 4.90)
**OCCUPATION CATEGORY^&^**								
Office worker	17	120,909	1.41	78	36,465	21.4	14.9(8.84, 25.3)^***^	6.60(3.13, 13.9)^***^
Laborer	29	71,197	4.07	70	36,686	19.1	4.68(3.03, 7.22)^***^	1.34(0.64, 2.81)
Other	8	21,707	3.69	35	13,174	26.6	6.91(3.20, 14.9)^***^	1.82(0.56, 5.93)
**COMORBIDITY^§^**								
No	36	174,670	2.06	11	9,715	11.3	5.44(2.77, 10.7)^***^	4.31(2.17, 8.53)^***^
Yes	18	39,142	4.60	172	76,610	22.5	4.82(2.96, 7.83)^***^	2.26(1.34, 3.79)^**^
**FOLLOW-UP PERIOD**								
≤1	10	63,739	1.57	45	31,029	14.5	9.57(4.82, 19.0)^***^	7.19(2.97, 17.4)^***^
2–3	15	7,159	21.0	62	4,190	148.0	7.11(4.04, 12.5)^***^	2.38(0.95, 5.94)
>3	29	116,748	2.48	76	42,672	17.8	7.16(4.66, 11.0)^***^	2.32(1.26, 4.26)^**^

*CI, confidence interval; HR, hazard ratio; PY, person-years*.

# *Incidence rate per 10,000 person-years*.

$ *Multivariable analysis included age, monthly income, urbanization level, occupation category, frequency of medical visits/per year, and comorbidities of depression, alcohol-related illness, anxiety, mental disorders, insomnia, acute pancreatitis, psychalgia, drug abuse, chronic obstructive pulmonary disease, diabetes mellitus, cardiovascular disease, and hyperlipidemia*.

† *New Taiwan Dollar (NTD), 1 NTD is equal to 0.03 USD*.

‡ *Urbanization level was determined by dividing the population density of residential areas into 4 levels, with level 1 being the most urbanized and level 4 the least urbanized*.

& Other occupation categories included those who were primarily retired, unemployed, and low-income populations;

§ *Individuals with schizophrenic, depression, alcohol-related illness, anxiety, mental disorders, insomnia, acute pancreatitis, psychalgia, drug abuse, chronic obstructive pulmonary disease, chronic kidney disease, diabetes mellitus, cardiovascular disease, and hyperlipidemia were classified as being in the comorbidity group*.

* *P < 0.05*,

** *P < 0.01*,

*** *P < 0.001*.

Table 4 shows the incidence and HRs of different types of suicide behavior for individuals with and without CP. Compared to individuals without CP, patients with CP tended to adopt suicide methods involving liquid or solid poisoning (aHR = 3.40, 95% CI = 1.91–6.05). Moreover, liquid or solid poisoning and cutting/piercing were the most common behaviors adopted by the CP cohort for suicide attempt.

**Table 4:** Incidence and hazard ratios of different types of suicide behavior between individuals with and without chronic pancreatitis.

	**Chronic pancreatitis**					
	**No**		**Yes**			
**Outcome**	**Event**	**Rate^#^**	**Event**	**Rate^#^**	**Crude HR (95% CI)**	**Adjusted HR^$^ (95% CI)**
Liquid or solid poisoning	33	1.54	117	13.6	8.57(5.82, 12.6)^***^	3.40(1.91, 6.05)^***^
Charcoal burning and poisoning by gases	3	0.14	11	1.27	9.15(2.55, 32.9)^***^	3.13(0.48, 20.4)
Hanging	1	0.05	5	0.58	11.0(1.29, 94.2)^*^	5.31(0.28, 100.3)
Cutting/piercing	7	0.33	36	4.17	12.9(5.73, 29.0)^***^	2.72(0.76, 9.75)
Jumping from high places	2	0.09	4	0.46	4.80(0.88, 26.3)	0.07(0.003, 1.64)
Others	8	0.37	10	1.16	3.13(1.23, 7.94)^*^	0.16(0.02, 1.28)

*CI, confidence interval; HR, hazard ratio; PY, person-years*.

# *Incidence rate per 10,000 person-years*.

$ *Multivariable analysis included age, monthly income, urbanization level, occupation category, frequency of medical visits/per year, and comorbidities of depression, alcohol-related illness, anxiety, mental disorders, insomnia, acute pancreatitis, psychalgia, drug abuse, chronic obstructive pulmonary disease, diabetes mellitus, cardiovascular disease, and hyperlipidemia*.

* *P < 0.05*;

*** *P < 0.001*.

After considering the competing risk of death, we observed that the CP cohort had a significantly higher risk of suicide attempt than did the non-CP cohort (adjusted SHR = 2.44, 95% CI = 1.53–3.88) (Table 5).

**Table 5:** Subhazard ratio (SHR) of suicide attempt for chronic pancreatitis cohort and non-chronic pancreatitis cohort estimated using competing-risks regression models.

	**Competing-risks regression models**	
	**Chronic pancreatitis**	
	**No**	**Yes**
**SUICIDE**		
Crude SHR (95% CI)	1(Reference)	8.31 (6.25, 11.0)^***^
Adjusted SHR^†^ (95% CI)	1(Reference)	2.44 (1.53, 3.88)^***^

*Crude SHR, relative subhazard ratio*.

*Adjusted SHR^†^, multivariable analysis*.

*Including all statistically significant risk factors in univariable Cox model*.

*** *P < 0.001*.

Table 6 indicates that the suicide-related fatality after attempting suicide was slightly higher in patients with CP than in those without CP (24.0 vs. 18.5%), but the difference was not statistically significant (aHR = 1.21, 95% CI = 0.39–3.78).

**Table 6:** Suicide-related fatality (completed suicide) of patients with chronic pancreatitis compared with those without chronic pancreatitis.

	**Chronic pancreatitis**	
	**No**	**Yes**
Suicide attempt (N)	54	183
Death (N)	10	44
Fatality rate (%)	18.5	24.0
Mean followed time ±*SD* (years)	2.88 ± 2.46	3.12 ± 2.65
Crude HR (95% CI)	1 (Reference)	1.52 (0.76, 3.01)
Adjusted HR^†^ (95% CI)	1 (Reference)	1.21 (0.39, 3.78)

*HR, hazard ratio*.

† *Multivariable analysis included age, monthly income, urbanization level, occupation category, frequency of medical visits/per year, and comorbidities of depression, alcohol-related illness, anxiety, mental disorders, insomnia, acute pancreatitis, psychalgia, drug abuse, chronic obstructive pulmonary disease, diabetes mellitus, cardiovascular disease, and hyperlipidemia*.

## Discussion

Findings from this administrative database revealed that CP was recorded at higher rates in men (82.6%) and in patients younger than 49 years (62.8%; mean age: 48.6 ± 15.2 years). Furthermore, alcohol-related illness and a history of acute pancreatitis were the most common comorbidities in patients with CP. All these findings may reflect the possibility of alcohol consumption being the main cause of CP in our cohort, although the NHIRD does not provide information pertaining to lifestyle and dietary habits. The causal relationship between CP and mental disorders has not been mentioned in the literature, but MRI previously showed that altered brain microstructures in patients with CP and altered brain areas (including the amygdala, cingulate cortex, insula, prefrontal cortex, and secondary sensory cortex) may be related to the development of mental disorders [32]. In addition, insomnia may be related to chronic pain and alcohol dependence in patients with CP [33]. The circular and multiplicative relationship between CP, depression, and substance use disorders has been evidenced in the literature, and biopsychosocial support is important in ameliorating this vicious cycle [34].

Our multivariable analysis indicated that the independent risk factors for suicide attempt were CP, lower urbanization level, depression, and mental disorders (Table 2). Our findings are consistent with the literature in demonstrating that individuals living in areas of lower urbanization levels (mostly farmers or those who are considered to be socioeconomically inferior) usually are more likely to attempt suicide [35]. Depression is the most common psychiatric disorder affecting people who die by suicide, because ~50–60% of patients who die by suicide have a history of depression [36]. Therefore, identifying individuals with depression is important to reducing suicide rates. Consistent with the literature, mental disorders have been reportedly increased the risk of suicide attempt through a general psychopathology liability in a population-based study [37]. Whereas we admit that alcohol-related illness cannot completely stand for alcoholism, and this may be the reason for no association between alcohol-related illness and suicide attempt. Otherwise, alcohol is among the major risk factors for suicide and it is associated with suicide, chronic disease, and unintentional injuries [38].

Our Cox proportional hazard regression results (Table 2) reveal a close association between CP and suicide attempt after adjustment for age, monthly income, urbanization level, occupation category, frequency of medical visits/per year, and comorbidities of depression, alcohol-related illness, anxiety, mental disorders, insomnia, acute pancreatitis, psychalgia, drug abuse, chronic obstructive pulmonary disease, diabetes mellitus, cardiovascular disease, and hyperlipidemia. The contribution of CP to the age-specific relative risk of suicide attempt was greater in younger patients, although it was non-significant in elderly patients. In addition, the contribution of CP to the relative risk of suicide was higher in patients without comorbidities than in patients with comorbidities (Table 3). The risk of suicide attempt increased in the CP cohort in line with the incremental follow-up duration after CP diagnosis, even though the follow-up duration was shorter in this cohort (Figure 1). Furthermore, we observed that the CP cohort consistently exhibited a higher risk of suicide, after considering the competing risk of death (Table 5). All findings in this observational study support an increased risk of suicide attempt after CP diagnosis although the suicide-related fatality was not significantly greater in the CP cohort (Table 6).

Among the suicide behaviors, poisoning and cutting/piercing are regarded as low-lethal methods; but charcoal burning and poisoning by gases, hanging, and jumping from high places belong to the high-lethal methods [39]. Compared with those aged ≥65 years, patients aged between 20 and 49 years were observed to be less likely to choose a highly lethal method when attempting suicide [40, 41]. Since liquid or solid poisoning and cutting/piercing were the most common suicide behaviors adopted by the CP cohort, with most patients aged younger than 49 years in our study, our findings can explain why the suicide-related fatality was not greater in the CP cohort. Chen et al. reported that the suicide mortality was greater in patients with pancreatic diseases among the Taiwanese with mental health disorders in the literature [26]. However, it should be noted all the population had previous psychiatric diagnoses and the case cohort included pancreatic diseases rather than CP. Furthermore, the etiologies of pancreatic diseases were more heterogeneous in the literature and, by the contrast, alcohol was the most common cause of CP in our study [26]. Finally, rather than suicide-related mortality as the endpoint in the literature, the endpoint of our study was suicide-related fatality.

The common methods adopted for suicide attempt in the frequency of order in Taiwan are poisoning, cutting/piercing, charcoal burning and poisoning by gases, hanging, and jumping from high places for men; whereas those for women are poisoning, cutting/piercing, charcoal burning and poisoning by gases, jumping from high places, and hanging for women [1]. According to our findings, providing enhanced suicide preventive programs is important for younger and middle-aged patients with CP. The top three methods adopted for suicide attempt in our CP cohort were similar to those reported for men in the literature since most of CP patients were men (82.6%). The common methods adopted for completed suicide in the frequency of order in Taiwan are poisoning, hanging, cutting/piercing, jumping from high places, and charcoal burning and poisoning by gases for men; whereas those for women are poisoning, hanging, jumping from high places, charcoal burning and poisoning by gases, and cutting/piercing for women [1]. Restriction of the sources of poisons or withdrawal of more toxic substances, such as pesticides and barbiturates, and reducing concentration of caffeine tablets have been demonstrated to be capable of reducing suicide incidence [4]. Moreover, detoxification of domestic gas and introducing catalytic converters in car are also effective in preventing suicide [42]. Nevertheless, restriction of the medium diffusion of a painless way of self-killing can reduce charcoal burning-related suicide attempt [43]. The financial status can also affect the rate of suicide attempts in Taiwan, such as higher suicide rate around 1999–2003 due to economic stagnation and increased suicide rate with incremental unemployment rate [44]. Education of the physicians should be enhanced since the rate of long-term usage of narcotic analgesic for CP is low in Taiwan from 2003 to 2012 [21]. The high rates of alcohol-related illness and psychiatric comorbidity in our CP cohort may reflect the importance of psychiatric referral for alcohol abstaining, prescription of anti-depressants or anti-anxiety drugs, and psychological support for suicide prevention. In addition, depression, anxiety, chronic pain, and drug abuse are independently associated with higher rates of suicide attempts [4]. From 2004 to 2013 in Taiwan, the suicide rate of men has much increased at age 30–60, with peak at age more than 85; whereas the suicide rate of women has flat mode at age 30–60, with upward-sloping peak at age 80–84 [44]. Living in an extended family can be protective of suicide and lacking resources to deal with marital and family problems is the main reason for the increased rate of suicide in women [45]. Unemployment and deprivation of economic domination in the family will increase the suicide rate of men at age 50–59, whereas the suicide rate in the middle-aged men are high in the divorced individuals [46, 47]. Therefore, the suicide prevention requires the multipartite cooperation between the society, family, and the individual.

Although no population-based cohort studies have explored the association between CP and suicide, we postulate the possible pathophysiological mechanisms involved in the increased risk of suicide attempt after CP diagnosis. CP may directly contribute to suicide through the following factors: physical factors such as chronic pain; psychological factors such as depression and guilt associated with alcohol use; and social factors such as disability and withdrawal from social or family activities or unemployment [19]. Moreover, alcohol consumption, the most common etiology of CP, may increase the risk of suicide by enhancing the secretion of serotonin and γ-aminobutyric acid and impairing cognitive functioning [38, 48]. A previous meta-analysis suggested that alcohol abuse significantly increases the risk of suicidal ideation, suicide attempts, and completed suicide [49]. The contribution of CP to the relative risk of suicide attempt was higher in patients without comorbidities than in patients with comorbidities even though the absolute risk of suicide attempt was greater in those with comorbidity (Table 3), and these findings may mean that CP per se or other possible non-mentioned CP-associated confounding factors may help contribute to the increased risk of suicide attempt. Not only alcohol-related illness, psychiatric or mental disorders, and physical comorbidities are common in our CP cohort, but also depression, anxiety, chronic pain, drug abuse, and physical comorbidities are independently associated with higher rates of suicide attempts [4]. Therefore, it requires more study to clarify whether the association between CP and suicide attempt is an epiphenomenon or a causal relationship.

Our study has several advantages. First, this is the first population-based cohort study conducted on the association between CP and suicide using a longitudinal database with a 12-year observation period in a large cohort of 1,000,000 residents in Taiwan. Second, the government-monopolized NHI program covers more than 99.5% of the residents in Taiwan; therefore, we believe that our findings of an association between CP and suicide are representative of the actual situation in Taiwan. Finally, the suicide-related fatality was found to be slightly higher in patients with CP than in patients without CP, but the result not statistically significant. However, the most common methods used by patients with CP to attempt suicide were liquid or solid poisoning, cutting/piercing, and charcoal burning and poisoning by gases. Our study could therefore be used as a reference to determine appropriate strategies for suicide prevention in patients with CP.

This study has several limitations. First, information on living environment and lifestyle is not registered in the NHIRD, and we used a diagnosis of alcohol-related illness and chronic obstructive pulmonary disease in place of using alcohol drinking habits and smoking, respectively. Moreover, the information of marital status or living alone is unavailable although the degree of social support is a risk factor for suicide. Although patient occupations were only categorized as official worker, laborer, or other occupation (which included those who were retired, unemployed, and those who had low-incomes), we found that suicide attempt was unrelated to monthly income or occupation. We classified the urbanization level based on the population density of the residential area (people/km^2^), and a greater association was found between urbanization and suicide at lower urbanization levels. However, the association between CP and suicide remained significant, irrespective of monthly income, occupation, or urbanization level (Table 3). Second, comorbidities might change during the course of follow-up, but the pre-existing comorbidities were redefined as long as they existed prior to the endpoint. However, the place of residence and socioeconomic status sometimes could not be updated in time. Therefore, the outcome of suicide attempt or completed suicide might be skewed. Third, we could not review individual records to validate diagnosis code accuracy, but all insurance claims and diagnosis codes would have been statutorily audited based on national guidelines. The NHI program is administered by the Taiwanese government, and medical providers face administrative sanction and financial penalties if diagnostic claims do not agree with the standard diagnostic criteria used for medical reimbursement. Moreover, the validity of administrative claims data between claims records in NHIRD and patient self-reports in the Taiwan National Health Insurance Interview Survey has been described in the literature with substantial concordance for the diagnosis of common chronic diseases, medication and health system utilization [50]. Fourth, alcohol might be the most common etiology of CP in our study, because alcohol-related illness was the most common comorbidity in the CP cohort. Multivariable analysis showed that CP was associated with suicide attempt, after adjustment for possible confounding factors, although we could not ascertain the actual etiology of CP. Finally, the attempting suicide rate could have been underestimated in the non-CP cohort if the behavior did not cause injury or the patient did not seek medical help.

We conclude that our population-based cohort study shows a close association between a diagnosis of CP and subsequent suicide attempt. Compared with the non-CP cohort, the suicide-related fatality was higher in the CP cohort, although this result was not statistically significant. Suicide is based on a complex interrelationship between psychological, biological, and social factors. Thus, detecting populations at a high risk of suicide attempt is important to preventing this behavior. In addition to endocrine and exocrine insufficiencies and chronic pain suffered by patients with CP, our findings necessitate surveying and providing psychological support to patients with CP, as well as providing public information on appropriate suicide prevention strategies.

## Author contributions

All authors listed have made a substantial, direct, and intellectual contribution to the work, and approved it for publication.

### Conflict of interest statement

The authors declare that the research was conducted in the absence of any commercial or financial relationships that could be construed as a potential conflict of interest.

## Abbreviations

CP: chronic pancreatitis
QOL: quality of life
aHR: adjusted hazard ratio
CI: confidence interval
NHI: National Health Insurance
NHIRD: National Health Insurance Research Database
NHRI: National Health Research Institutes
ICD-9-CM, International Classification of Diseases, Ninth Revision: Clinical Modification
SDs: standard deviations
HRs: hazard ratios
SHR: subhazard ratio
MRI: magnetic resonance imaging.
